# Primary Jejunal Melanoma Presenting as Widespread Metastases: Clinicopathological and Embryological Perspectives—A Case Report

**DOI:** 10.1155/cris/8003106

**Published:** 2026-04-24

**Authors:** Mahi Balcı

**Affiliations:** ^1^ Department of Pathology, Faculty of Medicine, Kırıkkale University, Kırıkkale, Türkiye, kku.edu.tr

**Keywords:** case report, disseminated melanoma, malignant melanoma, neural crest cells, primary jejunal melanoma, small intestine melanoma

## Abstract

Primary malignant melanoma of the small intestine is an exceptionally rare and diagnostically challenging condition, largely due to the predominance of metastatic involvement in the gastrointestinal (GI) tract. We report the case of a 71‐year‐old male presenting with a large, pigmented jejunal mass accompanied by multiple distant metastases in the liver, adrenal glands, and lungs. Histopathological and immunohistochemical analyses performed on the jejunal mass confirmed the diagnosis of malignant melanoma. Although systemic metastases were present, thorough clinical examination revealed no evidence of cutaneous, ocular, or mucosal primary lesions elsewhere, supporting the jejunum as the primary mucosal site of origin. This report discusses the embryological background, diagnostic challenges, and clinical significance of this rare entity. This case highlights that primary small bowel melanoma, although extremely rare, should be considered in patients presenting with intestinal lesions and disseminated metastases in the absence of an identifiable cutaneous primary lesion.

## 1. Introduction

Malignant melanoma is an aggressive neoplasm derived from melanocytes, which are normally found in the skin, uveal tract, leptomeninges, and certain mucosal sites. The gastrointestinal (GI) tract, particularly the small bowel, is a common site for metastatic melanoma, but primary melanoma arising from the small intestinal mucosa is exceptionally rare. Fewer than 50 cases have been reported in the English‐language literature, and their existence remains controversial [1].

The main diagnostic challenge lies in excluding an occult or regressed primary cutaneous, ocular, or mucosal lesion, as melanoma is known for its capacity for spontaneous regression [2]. Strict criteria, such as those proposed by Blecker et al. [3], require the absence of melanoma elsewhere and histological evidence of adjacent intramucosal melanocytic proliferation. However, even these criteria may not definitively rule out a regressed primary.

The embryological basis for primary intestinal melanoma has long been debated. While earlier hypotheses invoked aberrant migration of neural crest cells (NCCs) or APUD cell differentiation, recent lineage tracing studies have demonstrated that Schwann cell precursors (SCPs) associated with peripheral nerves can give rise to melanocytes postnatally. This finding offers a plausible mechanism for the presence of melanocytes—and thus primary melanoma—within the gut wall [4].

Herein, we report a case of presumed primary jejunal melanoma presenting with widespread metastases to the liver, adrenal glands, and lungs in a 71‐year‐old male, with no identifiable primary lesion elsewhere. This case is notable because the jejunal tumor was the initial and dominant manifestation, and the patient had a history of progressive peripheral neuropathy, which may be relevant in light of Schwann cell plasticity. We discuss the clinicopathological features and embryological perspectives to highlight the diagnostic complexities of this rare entity.

## 2. Case Presentation

A 71‐year‐old male patient presented with a history of rectal bleeding 2 years prior, initially diagnosed as anal fissure and hemorrhoids. Over the past year, he experienced numbness in his hands and feet and was diagnosed with cervical disc disease. One month before admission, he developed acute abdominal pain. On physical examination, the abdomen was distended with diffuse tenderness, and bowel sounds were decreased.

**Figure 1:**
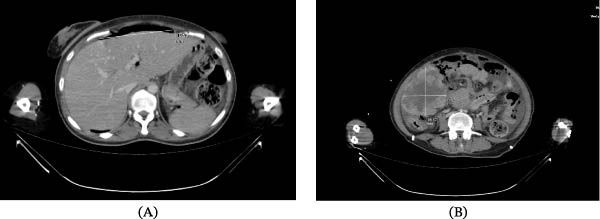
(A) Contrast‐enhanced axial abdominal computed tomography (CT) demonstrating free intraperitoneal air (arrow). (B) A large heterogeneous subhepatic mass with central necrosis measuring ~10 cm × 8 cm (arrows).

Contrast‐enhanced abdominal computed tomography (CT) demonstrated free intraperitoneal air anterior to the liver, consistent with GI perforation (Figure 1A). In addition, a large subhepatic mass measuring ~10 cm in greatest dimension, with central necrotic areas, was identified, suggestive of metastatic disease (Figure 1B). Free intra‐abdominal fluid was also present. Thoracic CT revealed multiple pulmonary nodules, the largest measuring ~5 cm in diameter.

**Figure 2:**
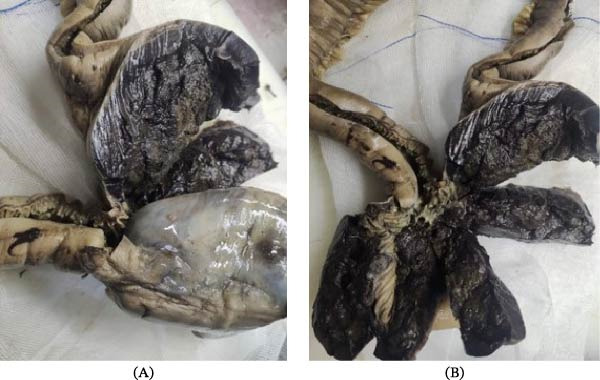
(A) Dilated jejunal segment showing a black‐colored tumor mass and (B) dilated jejunal segment showing a black‐colored tumor mass.

Due to the clinical findings suggestive of intestinal perforation, emergency partial small bowel resection was performed. Macroscopic examination revealed a 13 cm segment of jejunum with marked dilation caused by a black‐colored tumor infiltrating all layers of the bowel wall (Figure 2A,B).

**Figure 3:**
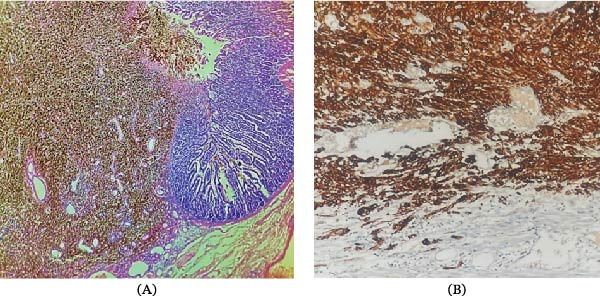
(A) Ulcerated pigmented tumor mass infiltrating the mucosa and submucosa with adjacent normal intestinal mucosa (H&E, ×100) and (B) strong cytoplasmic immunoexpression of HMB‐45 in tumor cells (×400).

Microscopic analysis demonstrated ulcerated mucosa infiltrated by atypical epithelioid and spindle‐shaped cells with heavy pigmentation extending to the serosa (Figure 3A). Immunohistochemical analysis revealed strong positivity for HMB‐45 and Melan‐A, confirming malignant melanoma (Figure 3B). Comprehensive dermatological and ophthalmological examinations revealed no evidence of a primary cutaneous or ocular melanoma. Following surgery, the patient was referred to the oncology department for further evaluation; however, he declined further oncological treatment and subsequently died during the follow‐up period.

## 3. Discussion

Malignant melanoma of the small intestine is a rare and diagnostically challenging entity, primarily because melanomas more commonly metastasize to the GI tract rather than arise there primarily. Autopsy studies have demonstrated GI involvement in over 60% of patients with disseminated melanoma; however, clinical detection of GI metastases remains uncommon (~2%) due to nonspecific symptoms such as abdominal pain, bleeding, and anemia [5–7].

Radiologically, small bowel melanoma may present with a variety of findings, including polypoid intraluminal masses, circumferential bowel wall thickening, intussusception, or signs of bowel obstruction. CT is the most commonly used imaging modality and may demonstrate hyperdense lesions related to melanin or hemorrhage, as well as associated metastatic disease in organs such as the liver, lungs, or adrenal glands [5]. In our case, contrast‐enhanced CT revealed a large subhepatic mass with central necrosis together with free intraperitoneal air suggestive of GI perforation, findings that prompted emergency surgical intervention.

Although our patient presented with multiple systemic metastases in the liver, adrenal glands, and lungs, thorough clinical evaluation, including detailed dermatological and ophthalmological examinations, revealed no evidence of primary melanoma at other sites. Diagnostic criteria for primary melanoma require the absence of melanoma at other primary locations and no history of excision of melanoma or atypical melanocytic lesions from the skin, retina, anal canal, or, less commonly, from sites such as the esophagus, penis, or vagina [3]. Additionally, Blecker et al.’s [3] criteria specify: (1) presence of a solitary mucosal lesion within the intestinal epithelium, (2) absence of cutaneous or ocular melanoma or atypical melanocytic lesions elsewhere, and (3) histological evidence of intramucosal melanocytic proliferation adjacent to the tumor. Our case fulfilled these criteria, supporting the diagnosis of primary jejunal melanoma. Although Blecker’s criteria were proposed in 1999, they remain the most widely accepted diagnostic framework for primary GI melanoma, as no updated consensus guidelines have been published since.

Embryologically, melanocytes arise from NCCs, a multipotent migratory population that differentiates into various lineages, including melanocytes and peripheral neurons [4, 8]. While melanoblasts have classically been thought to derive from trunk NCCs migrating via the dorsolateral pathway to skin and mucosa, recent studies reveal that SCPs, originating from ventrally migrating NCCs associated with peripheral nerves, also contribute substantially to melanocyte populations postnatally [8]. This dual origin broadens our understanding of melanocyte distribution and provides a plausible embryological basis for melanocytes and thus primary melanoma within the jejunal mucosa.

Interestingly, our patient had a history of progressive numbness in the hands and feet over the past year, radiologically attributed to cervical disc disease. However, this clinical finding may reflect peripheral nerve irritation or injury, which could be relevant in the context of melanocyte ontogeny. Notably, myelinating Schwann cells, although normally committed to glial fate, retain the plasticity to convert into pigment‐producing melanocytes under specific conditions such as nerve injury, where loss of nerve contact triggers reprograming [9]. To our knowledge, this is the first reported case of primary jejunal melanoma in a patient with concurrent peripheral neuropathy. While we cannot establish a causal relationship, the possibility that nerve injury‐related Schwann cell plasticity contributed to melanomagenesis in the gut wall is an intriguing hypothesis that warrants further investigation.

The differentiation of SCPs into melanoblasts is regulated by molecular signals, including Neuregulin‐1 (NRG1) and homeobox transcription factors such as Hmx1, which influence fate decisions between glial and melanocyte lineages. For example, NRG1 acting through ERBB3 receptors inhibits melanocyte fate, maintaining Schwann cell identity. Conversely, disruption of NRG1–ERBB3 signaling increases melanoblast differentiation [10]. Notably, the homeobox transcription factor HMX1 plays a crucial role in steering NCCs toward a neuronal fate. Experimental knockdown of Hmx1 results in a fate switch, increasing the number of MITF^+^ melanoblasts at the expense of neurons [11]. This suggests that in the absence of Hmx1, progenitor cells are more likely to adopt a melanocytic or glial identity.

We acknowledge that we did not investigate NRG1, ERBB3, or HMX1 expression in our patient’s tumor or nerve tissue. Therefore, the proposed link between peripheral neuropathy and melanocytic differentiation remains speculative. Future studies examining the expression of these molecules in primary GI melanomas and adjacent nerve fibers are needed.

From a clinical standpoint, melanomas arising from SCP‐derived melanocytes may exhibit distinct biological behaviors compared to those from dorsolateral NCCs, potentially influencing aggressiveness and therapeutic responses. This is particularly relevant for rare entities like primary jejunal melanoma, where lineage‐specific features might inform prognosis and personalized treatment.

Accurate diagnosis of primary intestinal melanoma is critical, as prognosis is generally poor despite surgical intervention, with 5‐year survival rates below 10% [6]. Our patient declined adjuvant therapy and died of progressive metastatic disease 3 months after surgery, reflecting this aggressive natural history.

## Funding

No funding was received for this research.

## Consent

No written informed consent has been obtained from the patient, as there is no patient‐identifiable information or images included in this case report.

## Conflicts of Interest

The author declares no conflicts of interest.

## Data Availability

The data that support the findings of this study are available from the corresponding author upon reasonable request.
